# Síndrome Coronária Aguda em Mulheres Idosas: A Inflamação Ataca Novamente

**DOI:** 10.36660/abc.20200092

**Published:** 2020-04-06

**Authors:** Bruno Rocha, Carlos Aguiar

**Affiliations:** 1 Departamento de CardiologiaHospital de Santa CruzCentro Hospitalar Lisboa OcidentalLisboaPortugalDepartamento de Cardiologia - Hospital de Santa Cruz - Centro Hospitalar Lisboa Ocidental, Lisboa - Portugal

**Keywords:** Aterosclerose, Inflamação, Nitrinas, Doença da Artéria Coronária, Síndrome Coronariana Aguda, Infarto do Miocárdio, Acidente Vascular Cerebral

A aterosclerose é o mecanismo patológico mais comum subjacente à doença arterial coronariana (DAC). Abrange tanto as síndromes coronárias agudas (SCA) quanto as síndromes coronárias crônicas (SCC). Tradicionalmente, o processo de formação de placa tem sido percebido como uma consequência do acúmulo de colesterol (particularmente a captação mediada por receptor sequestrador de lipoproteína de baixa densidade modificada), o qual leva ao crescimento contínuo de placa. O acúmulo na camada íntima subendotelial adicionalmente leva à estenose progressiva, ao fluxo sanguíneo reduzido e, eventualmente, à hipóxia tecidual. Em adição à isso, a oclusão espontânea de vasos trombóticos e os eventos embólicos podem constituir a via fisiopatológica comum para eventos cardiovasculares agudos maiores, a saber, infarto miocárdico (IM) e acidente vascular cerebral.^1^

Embora John Hunter tenha sido pioneiro na teoria inflamatória em 1794, foi somente em 1994 que desfechos piores em pacientes com SCA foram associados a níveis mais altos de proteína C reativa (PCR), e foi sugerido que a inflamação da placa pudesse ser responsável pela fissuração da placa. Dessa maneira, a inflamação finalmente foi associada à aterosclerose e à trombose. Além disso, foi subsequentemente demonstrado que a interleucina (IL) 1β, uma citocina pró-inflamatória, facilita a proliferação de células progenitoras hematopoiéticas por meio do metabolismo de glicose e colesterol, promovendo assim armadilhas extracelulares dos neutrófilos na placa crescente. De fato, o IM é acompanhado pela infiltração dos neutrófilos, que é fundamental para a regulação da inflamação.^2^

Atualmente, é comumente aceito que o ateroma é o resultado de um processo biológico dinâmico. Isto moderniza a visão antiga da placa como uma estrutura inerte de lipídios acumulados, substituindo-a pela visão de um centro vivo e turbulento de reações inflamatórias.

Apesar das investigações experimentais e dos ensaios clínicos iniciais com corticosteróides e antioxidantes terem sido decepcionantes, recentes ensaios clínicos de referência têm comprovado a assim chamada hipótese inflamatória de maneira convincente. No ensaio CANTOS,^3^ 10.061 pacientes com IM e PCR de alta sensibilidade (PCRas) elevada foram randomizados para receber 50, 150 ou 300 mg de canakinumab (um anticorpo monoclonal humano direcionado a IL-1β) ou placebo. O desfecho primário (morte cardiovascular, IM ou acidente vascular cerebral) foi significativamente reduzido no grupo de 150 mg de canakinumab em comparação com placebo (3,86/100 vs. 4,50/100 pessoas-anos; p = 0,02), em paralelo com uma redução da PCR. De fato, os pacientes que tiveram reduções maiores da PCRas obtiveram um benefício maior.^4^ O ensaio COLCOT^5^ incluiu 4.745 pacientes com IM recente (< 30 dias) e revascularização completa, os quais foram randomizados para uma baixa dose de colchicina (0,5 mg diariamente) ou placebo. O desfecho primário composto (morte cardiovascular, IM, acidente vascular cerebral, parada cardíaca ressuscitada ou hospitalização urgente devido a angina instável levando a revascularização) foi significativamente reduzido pela colchicina em comparação ao placebo (5,5% vs. 7,1%; p = 0,02), em paralelo com uma redução nos marcadores inflamatórios (a saber, PCRas).

É inegável que a inflamação desenvolve um papel nas doenças isquêmicas do coração, incluindo SCA e SCC. Entretanto, há várias perguntas intrigantes que ainda precisam ser elucidadas, incluindo as seguintes:

(1) Como detectamos a inflamação residual? Existem vários marcadores inflamatórios disponíveis, incluindo a PCRas, IL-1, IL-2, IL-6, IL-8, o fator de necrose tumoral α e a proteína quimioatrativa de monócitos-1 (MCP-1),^6^ apenas para citar alguns; em adição aos biomarcadores plasmáticos, as técnicas de imagem também podem oferecer outras medidas da inflamação (e.g., índice de atenuação de gordura perivascular determinado por angiotomografia coronária, a qual, tem sido demonstrado, melhora a previsão da mortalidade cardíaca além da avaliação atual).^7^ No entanto, ainda não tem sido claramente definido o parâmetro ideal para detectar a inflamação residual (facilmente medido e com boa relação custo-benefício);

(2) Quais são os pacientes que podem se beneficiar com tratamento anti-inflamatório? No ensaio CANTOS, os pacientes cuja PCRas diminuiu para < 2 mg/dL apresentaram uma redução de 25% de eventos cardiovasculares adversos maiores. No entanto, nós carecemos de um marcador decisivo para adequadamente selecionar os pacientes que terão maiores chances de se beneficiar com as terapias “anti-inflamatórias”, e deve ser observado que estas não são isentas de riscos (por exemplo, a canakinumab tem sido associada a celulite, colite pseudomembranosa e infecção ou sepse fatal). Portanto, nós precisaremos não somente refinar a detecção da inflamação residual, mas também descobrir novos medicamentos eficazes e seguros (ou redescobrir novas indicações para medicamentos existentes) para melhorar ainda mais os desfechos em pacientes com DAC;

(3) O risco residual é gerado apenas pela inflamação? Há evidências de que o risco residual pode ser devido à erosão e à fissuração da placa não relacionadas à inflamação sistêmica. Tem sido demonstrado que a vulnerabilidade da placa correlaciona-se com PCRas elevada e maior infiltração local de macrófagos (conforme medida por imagem de tomografia de coerência óptica intracoronariana),8 porém também foi demonstrado que a alteração do metabolismo do ácido hialurônico está associada à erosão da placa que pode não ser detectada pelos marcadores usuais de inflamação.9

Neste número do Jornal,^10^ Leocádio e seus colegas têm investigado o papel prognóstico da netrina-1 e da IL-1β em um estudo unicêntrico prospectivo incluindo 803 pacientes com SCA (333 mulheres, com uma idade média de 65 anos). Os autores têm verificado que níveis elevados de netrina-1 e IL-1β foram independentemente correlacionados com a mortalidade de todas as causas e/ou eventos cardiovasculares maiores em mulheres (mas não em homens) idosas no acompanhamento de dois anos. Curiosamente, em comparação aos homens, as mulheres idosas apresentaram uma prevalência mais alta de fatores de risco cardiovascular tradicionais, assim sugerindo que a inflamação pode ter desenvolvido um papel adicional decisivo na aterosclerose progressiva, aumentando os eventos significativos nesse subgrupo. Porém, essa hipótese precisará ser corroborada ainda mais, visto que estudos prévios têm consistentemente verificado um papel prognóstico de IL-1β indepdendente de sexo,^6^ e a netrina-1 não tem sido amplamente investigada na DAC. Além disso, os eventos foram raros em mulheres jovens (i.e., 3 mortes por todas as causas e 6 IM), assim impedindo qualquer conclusão definitiva. Todavia, merece avaliações futuras a ideia interessante de que esses biomarcadores possam resultar em intervenções personalizadas para pacientes cuidadosamente selecionados (e.g., mulheres na pós-menopausa).

A netrina-1 é um de cinco tipos de netrinas, de estrutura parecida às lamininas. Acredita-se que elas agem como um regulador dos neurônios e da migração celular durante o desenvolvimento. Podem estar envolvidos também na angiogênese (incluindo as vias de desenvolvimento do câncer), na lesão de isquemia-reperfusão e na aterosclerose. De fato, um estudo incluindo 180 pacientes com DAC e 79 controles sem DAC demonstrou que a netrina-1 (entre outros marcadores inflamatórios) foi mais efetivo que os biomarcadores clássicos no diagnóstico (número e gravidade de lesões) e na avaliação de riscos dos pacientes com DAC^11^ A IL-1β está entre as primeiras citocinas descritas, resultando da purificação de proteínas responsáveis para a indução da febre. Efeitos notáveis da IL-1β em tipos celulares diferentes incluem a ativação inflamatória de células endoteliais que participam no processo aterogênico. Além disso, tem sido demonstrado que a atividade da IL-1β é um preditor independente de mortalidade de todas as causas, SCA, fração de ejeção do ventrículo esquerdo menor e níveis mais altos de PCRas no estudo AtheroGene^12^ (registro prospectivo de 1.337 pacientes com DAC com SCA ou angina estável). Leocádio e seus colegas têm verificado um valor prognóstico potencial da elevação da netrina-1 e IL-1β no seu grupo de pacientes com SCA, particularmente em mulheres idosas, indicando um risco mais alto de eventos cardiovascular maiores, mesmo após ajustar para idade, tipo de SCA, diabetes mellitus, hipertensão e dislipidemia.^10^

Estudos futuros terão como foco a seleção adequada dos pacientes com DAC que mais poderão se beneficiar dos medicamentos “anti-inflamatórios” de modo eficaz e seguro. Dessa maneira, o papel do cardiologista que cuida desses pacientes pode eventualmente incluir o uso de ferramentas adequadas (e.g., biomarcadores plasmáticos) para identificar risco “residual” na prática clínica e reduzir mais os eventos cardiovasculares maiores por meio do combate à inflamação. O estudo em discussão7 sugere que a netrina-1 e a IL-1β podem ser úteis para a estratificação de risco cardiovascular em mulheres idosas.
