# Supplementary material for: Síndrome Coronária Aguda em Mulheres Idosas: A Inflamação Ataca Novamente
Source: Arq Bras Cardiol. 2020 Apr 6;114(3):515–7. [Article in Portuguese] doi: 10.36660/abc.20200092 (PMC7792726; doi:10.36660/abc.20200092)
Supplement: Translation

# Translation: Acute Coronary Syndrome in Elderly Women: Inflammation Strikes Again

**Authors:** Bruno Rocha, Carlos Aguiar

**Affiliations:** 1 PortugalDepartamento de Cardiologia - Hospital de Santa Cruz - Centro Hospitalar Lisboa Ocidental, Lisboa - Portugal

**Keywords:** Atherosclerosis, Inflammation, Netrins, Coronary Artery Disease, Syndrome Coronary Acute, Myocardial Infarction, Stroke

Atherosclerosis is the most common pathological mechanism underlying coronary artery disease (CAD), encompassing both acute coronary syndromes (ACS) and chronic coronary syndromes (CCS). Traditionally, the plaque formation process has been perceived as a consequence of cholesterol accumulation (particularly scavenger receptor-mediated uptake of modified low-density lipoprotein), leading to continuous plaque growth. Subendothelial intimal layer build-up further leads to progressive stenosis, reduced blood flow, and, eventually, tissue hypoxia. In addition, spontaneous thrombotic vessel occlusion and embolic events may constitute the common pathophysiological pathway for acute major cardiovascular events, namely myocardial infarction (MI) and stroke.^1^

Although John Hunter first pioneered the inflammatory theory in 1794, it was not until 1994 that worse outcomes in patients with ACS were linked to higher C-reactive protein (CRP) levels, and it was proposed that plaque inflammation could be responsible for plaque fissuring. Hence, inflammation was finally linked to atherosclerosis and thrombosis. Moreover, it was later demonstrated that interleukin (IL) 1β, a pro-inflammatory cytokine, facilitates hematopoietic progenitor cell proliferation through glucose and cholesterol metabolism, thus promoting neutrophil extracellular trap formation in the growing plaque. Indeed, MI is accompanied by neutrophil infiltration, which is paramount to inflammation regulation.^2^

Nowadays, it is commonly accepted that atheroma is the result of a dynamic biological process. This modernizes the old view, where plaque was seen as an inanimate mass of accumulated lipids, to the understanding of it as a turbulent, lively core of inflammatory reactions.

Despite initially disappointing experimental investigations and randomized controlled trials with corticosteroids and antioxidants drugs, recent landmark trials have convincingly proven the so-called inflammatory hypothesis. In the CANTOS trial,^3^ 10,061 patients with MI and elevated high-sensitivity (hs) CRP were randomized to 50, 150, or 300 mg of canakinumab (a human monoclonal antibody targeting IL-1β) or placebo. The primary outcome (cardiovascular death, MI, or stroke) was significantly reduced in the canakinumab 150 mg group in comparison to placebo (3.86/100 vs. 4.50/100 person-years; p = 0.02), in parallel with a reduction in hsCRP. In fact, patients who had greater reductions in hsCRP derived a greater benefit.^4^ The COLCOT trial^5^ enrolled 4,745 patients with a recent (< 30 days) MI and complete revascularization, who were randomized to low-dose colchicine (0.5 mg daily) or placebo. The primary composite outcome (cardiovascular death, MI, stroke, resuscitated cardiac arrest, or urgent hospitalization for unstable angina leading to revascularization) was significantly reduced by colchicine compared to placebo (5.5% vs. 7.1%; p = 0.02), in parallel with a reduction in inflammatory markers (namely hsCRP).

It is indisputable that inflammation plays a role in ischemic heart disease, including both ACS and CCS. However, there are multiple intriguing questions that have yet to be elucidated, including the following:

(1) How do we detect residual inflammation? There are several inflammatory markers available, including hsCRP, IL-1, IL-2, IL-6, IL-8, tumor necrosis factor α, and monocyte chemoattractant protein-1,^6^ just to name a few. In addition to plasma biomarkers, imaging may also offer measurements of inflammation (e.g., perivascular fat attenuation index determined by coronary computed tomography angiography, which has been shown to enhance cardiac mortality prediction over and above current assessment).^7^ Nonetheless, the optimal parameter for detecting residual inflammation (easily measured and cost-effective) has yet to be clearly defined;

(2) Which patients might benefit from anti-inflammatory treatment? In the CANTOS trial, patients whose hsCRP decreased to < 2 mg/dL had a 25% reduction in major adverse cardiovascular events; however, we lack a decisive marker to adequately select patients who may benefit the most from “anti-inflammatory” therapies, and it should be noted that these are not without risk (for instance, canakinumab was associated with cellulitis, pseudomembranous colitis and fatal infection or sepsis). Hence, we may need to be able not only to refine residual inflammation detection but also to discover new effective and safe drugs (or rediscover new indications for existing drugs) to further improve outcomes in patients with CAD;

(3) Is the residual risk driven solely by inflammation? There is evidence that residual risk may be due to plaque erosion and rupture unrelated to systemic inflammation. Plaque vulnerability has been shown to correlate with higher hsCRP and greater local macrophage infiltration (as measured by intracoronary optical tomographic coherence imaging),^8^ yet it has also been shown that alteration of hyaluronan metabolism is associated with plaque erosion that may not be detected by usual markers of inflammation.^9^

In this issue,^10^ Leocádio et al.^10^ have investigated the prognostic role of netrin-1 and IL-1β in a prospective single-center study enrolling 803 patients with ACS (333 women, with a mean age of 65 years). The authors have found that increased levels of netrin-1 and IL-1β were independently correlated with all-cause death and/or major cardiovascular events in elderly (> 60 years) women (but not in men) at 2-years of follow-up. Interestingly, compared to their counterparts, elderly women had a higher prevalence of traditional cardiovascular risk factors, hence suggesting that inflammation may have had an add-on decisive role for progressive atherosclerosis, increasing meaningful events in this subgroup. However, this hypothesis needs to be further corroborated, as previous studies have consistently found a prognostic role of IL-1β regardless of sex,^6^ and netrin-1 has not been widely investigated in CAD. In addition, events were scarce in young women (i.e., 3 all-cause deaths and 6 MI), hindering any definite conclusion. Nonetheless, whether these biomarkers may lead to tailored interventions in carefully selected patients (e.g., postmenopausal women) is an interesting concept worth further assessment.

Netrin-1 is one of five types of netrins, similar in structure to laminins. They are thought to act as a regulator of neurons and cell migration during development. They may also be involved in angiogenesis (including pathways in cancer development), anti-ischemia reperfusion injury and atherosclerosis. Indeed, a study enrolling 180 patients with CAD and 79 controls without CAD demonstrated that netrin-1 (amongst other inflammatory markers) was more effective than classical biomarkers in the diagnosis (number and severity of lesions) and risk assessment of patients with CAD.^11^ IL-1β is among the first described cytokines, resulting from the purification of proteins responsible for inducing fever. Notable effects of IL-1β on different cell types include inflammatory activation of endothelial cells participating in the atherogenic process. Furthermore, IL-1β activity has been shown to be an independent predictor of all-cause mortality, ACS, lower left ventricular ejection fraction, and higher hsCRP levels in the AtheroGene study^12^ (prospective registry of 1,337 CAD patients with ACS or stable angina). Leocádio and associates have found a potential prognostic value of increased netrin-1 and IL-1β in their cohort of patients with ACS, particularly in elderly women, indicating a higher risk of major cardiovascular events even after adjustment for age, type of ACS, diabetes mellitus, hypertension, and dyslipidemia.^10^

Future studies will focus on adequately selecting patients with CAD who may benefit most from “anti-inflammatory” drugs in an effective and safe manner. Hence, the role of the cardiologist caring for these patients may eventually include using adequate tools (e.g., plasma biomarkers) to identify “residual” risk in clinical practice and further reduce major cardiovascular events by tackling inflammation. The presented study^7^ suggests that netrin-1 and IL-1β may be of value in stratifying cardiovascular risk in elderly women.
